# Deep Brain Stimulation for Primary Refractory Tinnitus: A Systematic Review

**DOI:** 10.3390/brainsci14050452

**Published:** 2024-04-30

**Authors:** Landon Basner, Jasper V. Smit, Daniel M. Zeitler, Seth R. Schwartz, Katie Krause, Aiyush Bansal, Farrokh Farrokhi

**Affiliations:** 1 University of Washington School of Medicine, Seattle, WA 98195, USA; landonba@uw.edu; 2 Center for Neurosciences and Spine, Virginia Mason Franciscan Health, Seattle, WA 98101, USA; katie.krause@commonspirit.org (K.K.); aiyush.bansal@commonspirit.org (A.B.); 3 Department of Ear, Nose and Throat Surgery, Zuyderland Medical Center, 6419 PC Heerlen, The Netherlands; ja.smit@zuyderland.nl; 4 Head and Neck Surgery, Zuyderland Medical Center, 6419 PC Sittard, The Netherlands; 5 Department of Otolaryngology, Virginia Mason Medical Center, Seattle, WA 98101, USA; daniel.zeitler@virginiamason.org (D.M.Z.); seth.schwartz@virginiamason.org (S.R.S.)

**Keywords:** tinnitus, deep brain stimulation, neuromodulation, brain implant

## Abstract

Background: tinnitus is a common and often debilitating condition with limited evidence-based treatment options. Deep brain stimulation (DBS) is an approved treatment modality for certain neurological conditions; its experimental use as a treatment modality for severe tinnitus is novel and beginning to show promise. This systematic review focuses on the current evidence for the safety and efficacy of DBS for treatment of refractory tinnitus. Methods: a systematic search in PubMed and EMBASE was performed to identify peer-reviewed studies on DBS of non-cortical structures for the primary indication of tinnitus treatment. Three studies were identified as meeting these criteria, one of which had two related sub-studies. Results: seven patients with available data who underwent DBS for tinnitus were identified. DBS targets included nucleus accumbens (NAc), ventral anterior limb of the internal capsule (vALIC), caudate nucleus, and the medial geniculate body (MGB) of the thalamus. All studies used the Tinnitus Functional Index (TFI) as a primary outcome measure. DBS of the caudate was most commonly reported (n = 5), with a mean TFI improvement of 23.3 points. Only one subject underwent DBS targeting the NAc/vALIC (extrapolated TFI improvement 46.8) and one subject underwent DBS targeting the MGB (TFI improvement 59 points). Conclusions: DBS is a promising treatment option for refractory subjective tinnitus, with early data, from small patient cohorts in multiple studies, suggesting its safety and efficacy. Further studies with a larger patient population are needed to support this safety and efficacy before implementing this treatment to daily practice.

## 1. Introduction

Tinnitus is a highly prevalent disorder impacting an estimated 10–15% of American adults [1]. Currently, treatment modalities remain limited and with minimal positive impact. It is known that tinnitus becomes more prevalent with age [1], representing an emerging public health concern in light of an aging population. Tinnitus can have a significant impact on the quality of life of patients, with frequent comorbid psychological disorders such as depression and anxiety [2]. Additionally, it can have significant economic and social implications, with one study in the Netherlands [3] showing that tinnitus has a nationwide societal cost of €6.8 billion and a healthcare cost of €1.9 billion, while a British study [4] demonstrated a nationwide healthcare cost of £750 million per year. It is likely that similar trends exist in other countries, as the global prevalence of this condition is high, with a 2022 meta-analysis estimating that more than 740 million adults worldwide suffer from this condition [5].

Tinnitus can be categorized as objective, which represents a sound that may be audible to an external observer, such as turbulent blood flow, or subjective, which occurs without an acoustic stimulus. Subjective tinnitus is categorized as either primary idiopathic tinnitus associated with sensorineural hearing loss, or secondary tinnitus associated with an underlying cause, such as vestibular schwannoma or intracranial hypertension. Since treatment of most secondary tinnitus is best accomplished by addressing the underlying cause, this review focuses on primary subjective tinnitus. An important clinical consideration is that multiple types of tinnitus percepts may be concurrent in some patients, as described by Aldè et al. [6]

Unfortunately, treatment options for primary tinnitus remain limited and tinnitus is currently considered an incurable condition [1]. The American Academy of Otolaryngology–Head and Neck Surgery published a clinical practice guideline on tinnitus in 2014 which determined that many tinnitus treatments are ineffective and should not be used, including transcranial magnetic stimulation, dietary supplements, and all medical therapies. The only interventions with enough evidence to recommend were hearing aid evaluation, education and counseling, and cognitive behavioral therapy. Sound therapy, the current gold standard, had only enough evidence to be determined as an option. Thus, this patient population remains in need of an effective treatment for their condition.

Tinnitus has several leading theories behind its pathophysiology, most of which maintain that certain brain structures demonstrate hyperactivity because of inhibited input, often due to hearing loss [7,8,9]. Thus, deep brain stimulation (DBS), which can attenuate neural hyperactivity, was theorized to be an effective treatment modality. Furthermore, patients who had received DBS for other indications often reported improvements in the symptoms of their concurrent tinnitus [10].

The history of lesion analysis, animal studies, and reports of symptom reduction following DBS for other indications, have suggested DBS as a treatment for tinnitus [11,12,13,14]. DBS involves the electrical stimulation of electrodes, which are permanently implanted into the brain and connected by wires to an external pulse generator that emits an electrical signal with varying amplitudes and frequencies [15]. While the exact mechanism(s) of DBS remain unclear, one theoretical explanation is that pathologic neuronal activity is disrupted by the electrical stimulation [16]. For tinnitus, several non-cortical targets have been suggested for DBS therapy that show promise, making further evaluation a worthwhile pursuit. Furthermore, DBS has recently been used in early human trials for patients who are suffering from tinnitus, with promising efficacy.

This review serves to characterize the utility of DBS in the treatment of tinnitus in human studies, in efforts to advance further clinical trials of DBS for refractory tinnitus.

## 2. Materials and Methods

A literature search of PubMed and EMBASE was performed on 28 October 2023 to identify all studies using DBS to treat tinnitus. Timepoints were not predefined, but this search yielded publications from 2004 onwards. A search on clinicaltrials.gov was also conducted. Inclusion criteria were all studies looking at human invasive neuromodulation with the treatment effect of tinnitus symptom reduction based on subjective reports and validated scales. Exclusion criteria included all studies investigating cortical targets for neurostimulation, primary indications for DBS treatment other than tinnitus, and review articles. The following search terms were used:

### 2.1. PubMed

(“DBS”[All Fields] OR “deep brain stimulation”[All Fields] OR (“neuroprosthetic”[All Fields] OR “neuroprosthetics”[All Fields]) OR “brain implant”[All Fields]) AND (“tinnitus”[MeSH Terms] OR “tinnitus”[All Fields]) yielding 55 results.

### 2.2. EMBASE

(‘deep brain stimulation’ OR DBS OR ‘neuroprosthetic’ OR ‘neuroprosthetics’ OR ‘brain implant’) AND (‘tinnitus’), yielding 301 search terms.

A single author (LB) then performed a title and abstract review. A manual search through the results eliminated all duplicate entries. Automated deduplication was performed using Zotero. PRISMA methodology was followed during the inclusion and exclusion phases of the review.

### 2.3. Primary Outcomes

All studies used the Tinnitus Functional Index (TFI) [17] as a primary outcome measure, which is a standardized self-report questionnaire that was designed to maximize sensitivity in measuring treatment-related changes [17]. It should be noted that Dijkstra et al. [18] reported TFI on a scale of 0–10, while it is standard to use a scale of 0–100 when reporting TFI. Thus, for the data analysis, the TFI scores in the Dijkstra et al. manuscript were multiplied by 10 to maintain equivalency between papers, and will be referred to as the adjusted-TFI.

Two papers (Cheung et al. [19] and Dijkstra et al. [18]) used Tinnitus Handicap Inventory (THI) [20] as an additional outcome measure, while Devos et al. [21] used Tinnitus Questionnaire (TQ) [22]. Based on previous work using objective measures to quantify tinnitus handicap, there is excellent correlation between the THI and TQ, suggesting that they assess a similar tinnitus-related construct [23]. THI is a 25-question “self-report tinnitus handicap measure.” [20]. Likewise, TQ is a questionnaire defined by the Devos et al. study as classifying “the patient according to increasing degree of severity of the complaints: mild (up to 30 points), moderate (31–46 points), severe (47–59 points), or very severe (60–84 points).” [21,22]. Neither Dijkstra et al. [18] nor Devos et al. [21] specified the primary and/or secondary outcomes in their respective studies [18,21], so TQ and THI were included as an additional outcome in the current study in order to facilitate direct comparison between these results and those of Cheung et al. [19]. However, defining these as a primary or secondary outcome was intentionally avoided.

### 2.4. Secondary Outcomes

Other outcomes assessed by Devos et al. were anxiety and depression using the Hospital Anxiety and Depression scale, refs. [21,24] as well as hearing via audiometry [21]. Cheung et al. also reported outcomes using the Frontal Systems Behavior Scale, a tool used to assess frontal structure damage [25], EF score (executive function safety score), “a composite z-score from multiple neuropsychological tests”, and objective hearing assessments using standard audiometry, including pure tone thresholds and word recognition testing. Additionally, the study by Dijkstra et al. reported on scores using the Hamilton depression rating scale [26].

## 3. Result

**Figure 1:**
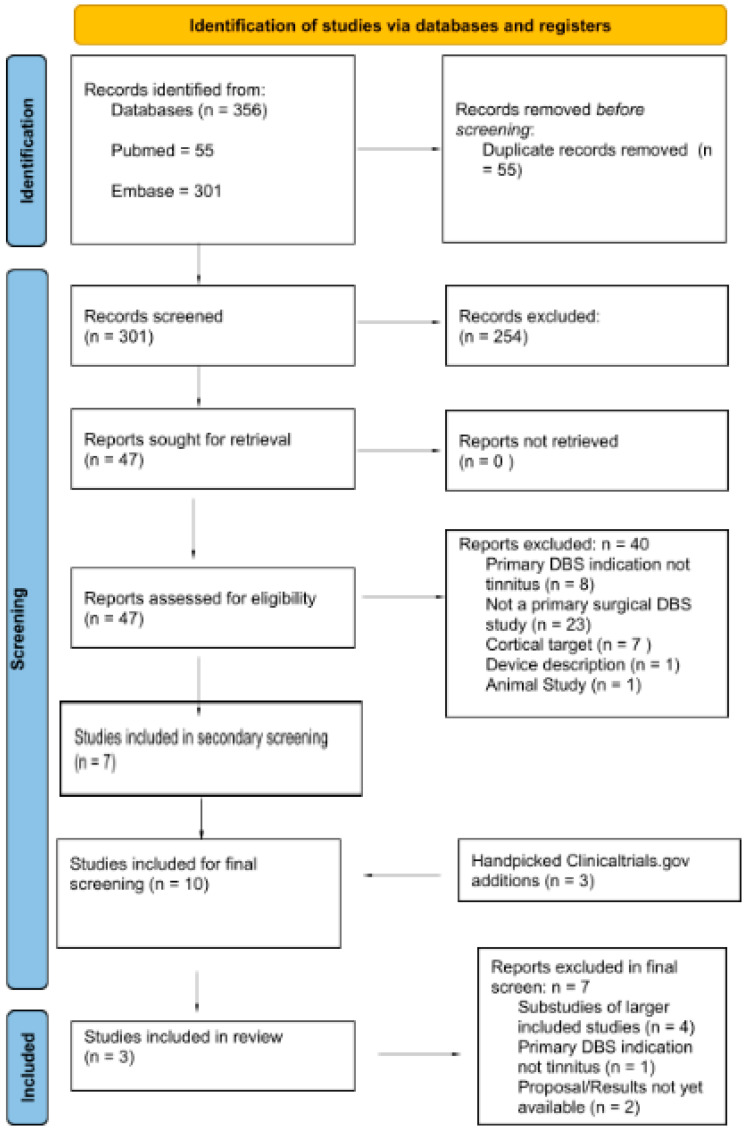
PRISMA diagram detailing the selection process for studies included in the review.

The search in PubMed yielded 55 articles, and the search in EMBASE yielded an additional 301 articles. There were 55 duplicate articles. There were a total of 47 articles that looked at human invasive neuromodulation with a treatment effect of tinnitus symptom change; of these, seven were non-cortical targeted and for the primary indication of tinnitus treatment. A search on clinicaltrials.gov revealed an additional three studies that were included in the analysis for a total of 10 articles. Of the ten remaining articles, the primary author (LB) performed a full text review and seven of the studies failing to meet inclusion and/or exclusion criteria were removed from final analysis. After this systematic approach, the final three studies were used for data extraction for cases for the final review (Figure 1). For these studies we identified the target for DBS, outcome measures of interest, device used, and complications. We pooled these cases into a table (Table 1) but due to the small numbers we were unable to perform a formal meta-analysis.

Of the three clinical trials on clinicaltrials.gov, two are actively recruiting and ongoing with estimates of six [21] and seven [27] patients each. Preliminary results for one patient in the former study have been reported [21]. One trial has concluded, with five patients completing the study [3]. Two case reports were included [18,21], one of which is the aforementioned preliminary case report of the n = 6 study being conducted at Maastricht University Medical Center (The Netherlands). Thus, seven patients in total have been identified who have received DBS for the primary indication of tinnitus treatment, and whose results have been published.

Of the studies reviewed, six out of seven patients had electrodes placed in striatal structures. Five of these patients had exclusive targeting of the striatum via placement in the caudate18, while the DBS lead tips in the Dijkstra et al. [18] study targeted a striatal structure (the NAc), with DBS leads also traversing the vALIC (a non-striatal structure), wherein two contact points of the lead were stimulated. Devos was the only investigator who exclusively selected non-striatal structures by targeting the medial geniculate body (MGB) of the thalamus [21].

The patient in the Dijkstra et al. trial did not have hearing status parameters formally described, but she did have hyperacusis at baseline, which emerged alongside her tinnitus following a unilateral cholesteatoma removal from the left ear. She did not have testing of pure-tone audiometry, speech audiometry, otoacoustic emissions, or auditory brainstem responses [18].

Cheung et al. assessed hearing changes following the DBS implantation using six different air-conduction noise frequencies, with no significant changes observed. Study patients did not have “hyperacusis, misophonia, and average air conduction of any 3 consecutive audiometric frequencies (0.5, 1, 2, 4, and 8 kHz) ≥ 56 dB in either ear”. Utilization of speech audiometry, otoacoustic emissions, and auditory brainstem responses were not considered [19]. Likewise, Devos et al. used pure-tone audiometry as an inclusion criterion, where, “Average pure tone thresholds for 1, 2 and 4 kHz” were “<60 dB for each ear” [16]. In the one patient from this study whose results had been reported, no hearing changes occurred [21]. Speech audiometry and auditory brainstem responses were also assessed, although these were not reported on their first patient case report. Otoacoustic emissions were not measured [21].

Per our review, targeted DBS of the MGB of thalamus resulted in a TFI reduction of 59 (Table 1). All patients had baseline TFIs greater than 60 (range, 61.6–89.2). Targeted stimulation of the NAc and vALIC resulted in an adjusted-TFI reduction of 46.8. The mean TFI reduction among the five patients receiving DBS in the caudate was 23.32 (range, 5.2–86.8). However, the largest effects were seen in DBS of the caudate, with reductions of 86.8 and 73.2 demonstrated in two patients. A baseline TFI greater than 50 was an inclusion requirement for participation in the Cheung et al. [19] clinical trial, while the trial [16] from which Devos et al. [21] was first reporting patient data had a TQ of greater than 47 as an inclusion requirement.

### Side/Adverse Effects

The patient in the Dijkstra et al. study reported mild forgetfulness following DBS [18]. Devos et al. reported that a patient experienced “transient tingling” of the contralateral body following increased DBS voltage [21]. Cheung et al. reported one serious adverse effect in a patient who attempted suicide, which was felt to be unrelated to DBS as the subject had been off stimulation for 2 months prior to the event [19]. All six patients in the Cheung et al. trial [19] experienced transiently worsening tinnitus, six had post-op incisional pain, four experienced post-op headache, three reported a pulling sensation at the internal pulse generator (IPG) site (bowstringing) [28], two experienced facial/neck tingling and lightheadedness/dizziness, two reported post-operative fatigue and sleep disturbances, two had worsened depression, and there was one report each of increased energy, post-operative nausea, and visual phantoms (Table 2). According to the authors, all of the reported adverse effects were temporary, with the exception of elevated electrode impedances in one participant [19]. These side effects are similar to those reported for other DBS indications [28,29,30,31,32,33].

## 4. Discussion

Subjective tinnitus is a heterogeneous condition wherein patients may experience different pitches, noises [34], and these may have the quality of being tonal vs. complex [34]. The diverse nature of tinnitus and its complex pathophysiology, without an easily identifiable aberrant lesion or structure, makes it a unique treatment challenge. The concurrent psychological symptoms often seen further challenge the treatment.

This review found, in a few cases, positive results on safety and efficacy in tinnitus reduction in different DBS targets. There is not yet a consensus on the optimal location for DBS for tinnitus. The MGB, the target chosen by Devos, was selected following successful DBS inhibition of tinnitus in a rat model [35]. Meanwhile, the caudate, the target of choice in the Cheung trial, was selected after reports of tinnitus resolution following infarction of the caudate [12,36], fMRI findings [37], and modulation of the caudate during DBS lead implantation in movement disorder patients [14,38]. With this in mind, it is interesting to note that the caudate is not traditionally considered a part of the auditory pathway [39]. Dijkstra et al. [18] largely based their decision to target NAc and vALIC on imaging and pathway data [18].

Dijkstra et al. [18] reported that placement of bilateral DBS leads in the NAc, alongside the vALIC, leading to significant reductions in symptomatology of severe tinnitus in a 41-year-old woman following cholesteatoma surgery. This ear surgery would have been an exclusion criterion for the trial [16] from which Devos was reporting, and probably also from the Cheung et al. trial [3]. Especially in the Dijkstra et al. study [18], it can be discussed whether primarily her depression was treated by DBS, thereby improving the burden of the tinnitus, or the tinnitus itself. Namely, the vALIC was earlier targeted by this group for depression; the NAc was targeted for obsessive–compulsive disorder. Cheung et al. [19] reported on five patients treated with DBS targeting the caudate nucleus, with a mean TFI improvement of 23.3, where three patients experienced clinically significant (defined as a greater than or equal to 13-point decrease in TFI) [19] improvement. THI had a mean improvement of 30.8, where four patients experienced clinically significant (defined as a greater than or equal to 20-point decrease in THI) [19] improvement (Table 3). A follow-up study was conducted on two of these patients, one of whom had gained considerable benefit and one of whom had no benefit. Both patients had at least one lead in the caudate tail (CT).

The Cheung et al. study initially identified nine specific regions of the striatum (one of which was the CT) and used MRI data from a larger cohort, representing a variety of hearing and tinnitus phenotypes, to determine caudate regions associated with an abnormal connectivity map. One DBS patient whose left lead was positioned in the CT and right lead was positioned in the dorsal medial caudate body of the striatum exhibited significant improvements. The personalized striatal networks of this patient showed correspondence to increased CT connectivity with “superior parietal lobe bilaterally, left temporal lobe along the superior/middle/inferior temporal gyrus, and the left ventral cerebellum”, while the right lead showed no correspondence in increased connectivity. The other DBS patient whose left lead was in CT and right lead was also in CT showed no improvements and only increased connectivity with “frontal regions and the brainstem” was seen. Importantly, the study points out, “personalized striatal network maps appear to be critical for effective treatment target selection” [17], as there may be some heterogeneity in tinnitus networks among candidates.

A further follow-up study [39] of the Cheung et al. clinical trial has also been published, wherein intraoperative mapping occurred in six patients, with DBS probes being placed at up to three locations bilaterally (+/− 5 mm anterior to posterior) in the caudate, or at the first caudate location where significant tinnitus loudness modulation occurred. A total of 20 locations (twelve left, eight right) were tested between the six patients, with five locations (on the antero–posterior axis) showing a decrease in tinnitus loudness, and four of these five locations being located more posteriorly in the caudate body [39].

Thalamic structures were one of the earliest sites of investigation for DBS treatment of tinnitus, where three of seven tremor patients with concurrent tinnitus reported symptom improvement following DBS, in a chart review conducted by Shi et al. [11] An auditory region of the thalamus, the MGB, was the target selected by Devos et al. in a 54-year-old male patient who was a participant in a larger study [16]. The patient reported significant improvements, per TFI score [21].

In the one patient who received stimulation of NAc and vALIC, only forgetfulness was seen as a side effect. [18] For the patients receiving caudate stimulation, all six patients experienced tinnitus that worsened when stimulation parameters were not optimal. Additionally, three patients experienced bowstringing and tingling. Sleep disturbances and worsening depression were each experienced by one patient, respectively [19]. In the MGB, no side effects other than transient tingling were reported [21].

In most cases, tinnitus is associated with hearing loss. This idea introduces us to a tinnitus theory known as “central gain”, which postulates that the loss of sensory input leads to hyperexcitation of auditory circuits [23]. However, tinnitus can also occur in patients without clinically significant hearing loss and is potentially linked to cochlear synaptopathy due to aging or noise exposure that is below the clinically detectable limit, however, this remains controversial [23]. A related theory, known as discordant theory, is associated with the dorsal cochlear nucleus (DCN) at the pontomedullary junction. This theory describes peripheral insults which generally damage outer hair cells more than inner hair cells, causing a downstream disinhibition of neurons in the DCN, which then increases spontaneous activity in the central auditory system [23]. This could explain the positive effect of DBS in the MGB, which is within the auditory pathway, since DBS tends to modify the pathological (‘tinnitus’) activity to a more regular pattern [40]. Tinnitus is frequently thought of in parallel to phantom pain [23] and, similarly to phantom pain phenomena, frontostriatal pathways may be involved. Per Rauschecker et al. [23], the ventromedial prefrontal cortex and the NAc of the striatum have been identified as playing a role in gating the significance of sensory information, as well as gating descending pathways.

The NAc is thus a possible site of stimulation and was the site selected by Dijkstra et al. [18] This site was chosen because of the role that the NAc plays in “emotional gating of the tinnitus stimulus”, alongside the fMRI observation captured by Rauscheker et al. that the NAc was especially hyperactive in patients with tinnitus [41].

Cheung et al.’s site selection [19] was also informed by an fMRI study [37], which demonstrated that the dorsal caudate had markedly and consistently elevated connectivity with the auditory cortex in tinnitus patients when compared to controls. This pathway was hypothesized to be necessary for perceiving auditory phantoms and thus modulating the pathway could inhibit this perception.

### Limitations

The small quantity of patient data that was identified was the most obvious limit of this study. Another limit of this study was that only one person performed screening, which could have increased subjectivity in the screening process.

In short, while multiple DBS targets for tinnitus exist, there is insufficient clinical data to definitively identify any of the trialed targets as superior.

## 5. Conclusions/Future Directions

While the total number of cases of reported DBS for tinnitus remains low, it appears to be a safe and viable treatment modality with considerable efficacy. Since only seven patients have reportable data, it is too early to recommend one specific target. It is also likely that other sites will be explored, as other targets have been investigated in animal models. Whether one site becomes standard for most patients, or whether individualized site determination proves superior is unclear at this time. Although there remain many unanswered questions around DBS for tinnitus, the limited findings summarized in this review show early promise.

## Figures and Tables

**Table 1:** All patients in table had bilateral lead placement, Dijkstra [18] and Devos [21] patients received Medtronic 3389 leads while Cheung patients received 3387 leads. * = per Cheung et al. [19], “in U01-02, in whom the lead in the left caudate was turned off; this particular participant preferred unilateral stimulation despite the fact that bilateral stimulation was well tolerated”; vALIC = ventral anterior limb of the internal capsule; adjusted-TFI was calculated by multiplying the Dijkstra-reported TFIs by 10—rationale for this is provided in our methods section. Percent reduction = ((Original value − Final Value)/Original value) × 100.

Patient/Study	Age/Sex	Starting TFI	Ending TFI	TFI Percent Reduction	Time to TFI Maximum Improvement	Site
41 F Dijkstra	41 F	74 (adjusted-TFI, wherein a value of 7.4 was reported)	27.2 (Adjusted-TFI, wherein a value of 2.72 was reported)	63.24%	9 mo	vALIC + NAc
54 M Devos	54 M	69	10	85.51%	12 mo	MGB of thalamus
38 F Cheung *	38 F	76.8	73.2	4.69%	18	Caudate nucleus
58 M Cheung (patient #3)	58 M	66.4	46	30.72%	16	Caudate nucleus
58 M Cheung (Patient #4)	58 M	61.6	42	31.82%	21	Caudate nucleus
37 F Cheung	37 F	75.6	5.2	92.86%	19	Caudate nucleus
62 M Cheung	62 M	89.2	86.8	2.69%	20	Caudate nucleus

**Table 2:** Side effects. This table is not meant to imply incidence of side effects, as these 3 studies had significant heterogeneity in methods of reporting side effects and an incidence calculation would likely be skewed by question-behavior effect. It is simply meant to indicate total instances of the reports of these side effects; 8 patients are reported upon for side effects, rather than the 7 who had tinnitus outcome measures reported upon, because Cheung et al. [19] included the drop-out patient in their side effects section.

Scheme 8	Instances Reported (from a Sample of 8 Patients)
Tingling of the body	n = 1
Post-op incisional pain	n = 6
Transiently worsened tinnitus	n = 6
Post-op headache	n = 4
Pulling sensation at IPG	n = 3
Face/neck tingling	n = 2
Lightheadedness/dizzy	n = 2
Post-op fatigue	n = 2
Sleep disturbance	n = 2
Worsened depression	n = 2
Increased energy	n = 1
Post-op nausea	n = 1
Visual phantoms	n = 1
Suicide ideation	n = 1
Mild forgetfulness	n = 1

**Table 3:** THI “The correlation between total scores of THI and TQ is 0.641 (*p* < 0.0001), indicating that they assess a similar tinnitus-related construct” [23]. Percent reduction = ((Original value − Final Value)/Original value) × 100.

Patient	Age/Sex	Starting THI/TQ	Ending THI/TQ	THI/TQ Reduction	Time to THI/TQ Max Improvement	Site
Dijkstra 1	41 F	76 (THI)	20 (THI)	73.68%	9 mo	vALIC + NAc
Devos 1	54 M	54 (TQ)	16 (TQ)	70.37%	12 mo	MGB of thalamus
Cheung 2	38 F	76	56	26.32%	18 mo	Caudate nucleus
Cheung 3	58 M	54	32	40.74%	16 mo	Caudate nucleus
Cheung 4	58 M	34	18	47.06%	21 mo	Caudate nucleus
Cheung 10	37 F	74	2	97.29%	19 mo	Caudate nucleus
Cheung 12	62 M	82	58	29.27%	20 mo	Caudate nucleus

## Data Availability

All data came from three clinical studies, which correspond to references [18,19,21] on our works cited page.
